# Gut microbiome dysbiosis is associated with lumbar degenerative spondylolisthesis in symptomatic patients

**DOI:** 10.1002/jsp2.70005

**Published:** 2024-10-10

**Authors:** Khaled Aboushaala, Ana V. Chee, Darbaz Adnan, Sheila J. Toro, Harmanjeet Singh, Andrew Savoia, Ekamjeet S. Dhillon, Catherine Yuh, Jake Dourdourekas, Ishani K. Patel, Rajko Vucicevic, Alejandro A. Espinoza‐Orias, John T. Martin, Chundo Oh, Ali Keshavarzian, Hanne B. Albert, Jaro Karppinen, Mehmet Kocak, Arnold Y. L. Wong, Edward J. Goldberg, Frank M. Phillips, Matthew W. Colman, Frances M. K. Williams, Jeffrey A. Borgia, Ankur Naqib, Stefan J. Green, Christopher B. Forsyth, Howard S. An, Dino Samartzis

**Affiliations:** ^1^ Department of Orthopedic Surgery Rush University Medical Center Chicago Illinois USA; ^2^ Center for Integrated Microbiome & Chronobiology Research, Rush Medical College, Rush University Medical Center Chicago Illinois USA; ^3^ Department of Internal Medicine Rush University Medical Center Chicago Illinois USA; ^4^ Research Unit of Health Sciences and Technology University of Oulu Oulu Finland; ^5^ Department of Radiology & Nuclear Medicine Rush University Medical Center Chicago Illinois USA; ^6^ Department of Rehabilitation Sciences The Hong Kong Polytechnic University Hong Kong China; ^7^ Department of Twins Research and Genetic Epidemiology King's College London UK; ^8^ Departments of Anatomy & Cell Biology and Pathology Rush University Medical Center Chicago Illinois USA

**Keywords:** degeneration, disc, dysbiosis, facets, imaging, microbiome, spondylolisthesis

## Abstract

**Background:**

Lumbar degenerative spondylolisthesis (LDS), characterized as degeneration of the intervertebral disc and structural changes of the facet joints, is a condition with varying degrees of instability that may lead to pain, canal stenosis, and subsequent surgical intervention. However, the etiology of LDS remains inconclusive. Gut microbiome dysbiosis may stimulate systemic inflammation in various disorders. However, the role of such dysbiosis upon spine health remains under‐studied. The current study assessed the association of gut microbiome dysbiosis in symptomatic patients with or without LDS.

**Methods:**

A cross‐sectional analysis within the framework of a prospective study was performed. DNA was extracted from fecal samples collected from adult symptomatic patients with (*n* = 21) and without LDS (*n* = 12). Alpha and beta diversity assessed differences in fecal microbial community between groups. Taxon‐by‐taxon analysis identified microbial features with differential relative abundance between groups. Subject demographics and imaging parameters were also assessed.

**Results:**

There was no significant group differences in age, sex, race, body mass index, smoking/alcohol history, pain profiles, spinopelvic alignment, and Modic changes (*p* >0.05). LDS subjects had significantly higher disc degeneration severity (*p* = 0.018) and alpha diversity levels compared to non‐LDS subjects (*p* = 0.002–0.003). Significant differences in gut microbial community structure were observed between groups (*p* = 0.046). Subjects with LDS exhibited distinct differences at the phylum level, with a significantly higher Firmicutes to Bacteroidota ratio compared to non‐LDS (*p* = 0.003). Differential relative abundance analysis identified six taxa with significant differences between the two groups, with LDS demonstrating an increase in putative pro‐inflammatory bacteria (*Dialister, CAG‐352*) and a decrease in anti‐inflammatory bacteria (*Slackia*, *Escherichia‐Shigella*).

**Conclusion:**

This study is the first to report a significant association of gut microbiome dysbiosis and LDS in symptomatic patients, noting pro‐inflammatory bacterial taxa. This work provides a foundation for future studies addressing the role of the gut microbiome in association with spine health and disease.

## Graphical abstract

This study is the “first” to report a significant association of gut microbiome dysbiosis and the association with lumbar degenerative spondylolisthesis in symptomatic patients, noting pro‐inflammatory bacterial taxa. This work provides a foundation for future work addressing the role of the gut microbiome in association with spine health and disease.

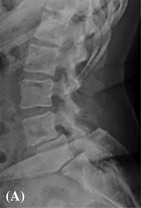

## 1 INTRODUCTION

Chronic low back pain is an aggressively debilitating condition that causes significant disability and financial burden worldwide.^1^ It is currently the leading cause of disability globally, yet most cases are nonspecific, and the etiology is difficult to isolate due to its multifactorial nature.^2^ Various population‐based cohorts have demonstrated that disc degeneration plays a significant role in the development of chronic low back pain.^3^, ^4^

**FIGURE 1:**
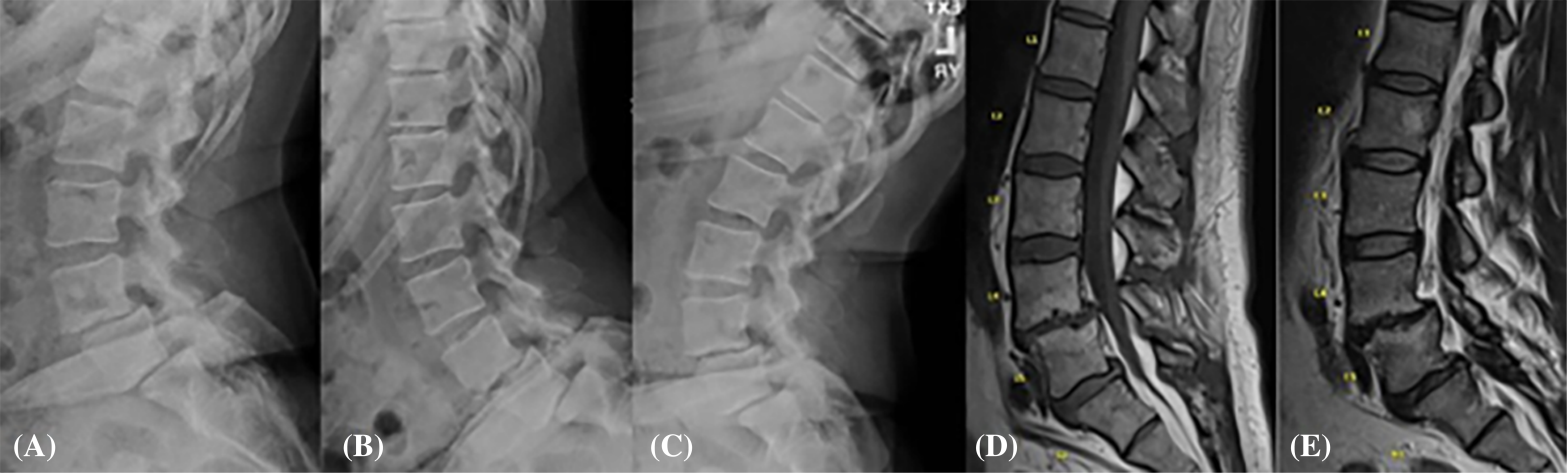
(A)–(C) Dynamic lateral lumbar plain radiographs and (D), (E) lumbar T1‐ and T2‐weighted MRI noting spondylolisthesis at L4‐L5 and L5‐S1 in a 65‐year‐old female.

Although the spectrum of disc degeneration can be wide, a secondary consequence may result in lumbar degenerative spondylolisthesis (LDS; Figure 1). LDS was first reported by Newman and Stone in 1955, based on a clinical, radiological study, and characterized as slippage of the vertebrae over an adjacent vertebra due to degeneration of the intervertebral disc and lumbar facet joints.^5^ Since then, numerous classification schemes of LDS have been reported, but are largely distinguished based on the following etiologies: isthmic, traumatic, pathologic, dysplastic, and degenerative. LDS is the most common in adults, increasing in risk in older age,^6^ commonly seen in patients above the age of 50, predominantly occurring in females (6:1 prevalence), and the L4–L5 spinal levels are mainly affected.^7^, ^8^, ^9^, ^10^, ^11^ Facet joint arthritis, increased ligamentous laxity predisposing to micro instability, and disc degeneration causing narrowing of the disc space are potential causes for the development of LDS impacting quality of life and interfering with daily function.^8^, ^12^, ^13^ Radiculopathy and neurogenic claudication, with or without low back pain, are common symptoms experienced by patients that may guide treatment options, including surgical stabilization.^14^

Extensive research has been conducted to help determine predisposing risk factors for the development of LDS. Age, sex, ethnicity, improper diet, physical activity levels, and bone density levels have all been implicated in the disease process. However, little is known with regard to the gut microbiome and its role in spinal health.^15^

Commonly referred to as the “hidden organ,” the gut microbiome refers to the ~100 trillion microorganisms including bacteria, fungi, and viruses that reside in the human intestine.^16^ While the origins of the microbiome and microbe isolation research date back to the 19th century, it was not until the 1980s that advances in computation and genomic sequencing technology broadened our evolving understanding of the vast diversity and classification of bacterial phylogeny.^17^ To date, most microbiome‐related research has focused on bacteria, with gut bacteria having known biological roles in digestion, metabolism, immunity, and metabolite synthesis.^18^ There continues to be evidence that “dysbiosis” or alterations in healthy resident gut bacteria are associated with numerous gastrointestinal and non‐gastrointestinal disease processes, including asthma, obesity, diabetes, inflammatory bowel disease, chronic kidney disease, and various neurodegenerative conditions.^16^, ^19^, ^20^, ^21^, ^22^

While animal models are extensively utilized in current microbiome research, various human studies have explored the possible link between changes in gut microbiota and musculoskeletal pathology.^22^ Wang et al.^23^ found that osteoporosis was associated with an increased proportion of Bacillota (formerly Firmicutes) and a decreased proportion of Bacteroidota compared to controls. Cardoneanu et al.^24^ discovered that decreased diversity of the gut microbiota was associated with ankylosing spondylitis. One study demonstrated that in an obese population, changes in fecal microbial community structure were correlated with back pain. For example, the relative abundance of bacteria from the genus *Adlercreutzia* was significantly associated with back pain.^25^ Such changes of the microbiome lend further discussion as to bacteria‐induced inflammation as attributed to a leaky gut or direct bacterial invasion of tissue that can lend to a cascade of pathological changes in time or an interaction of both pathways.

With respect to the spine and degenerative painful conditions, Albert et al.^26^ were instrumental in bringing forward the concept of an infectious etiology related to pathology. Their initial studies suggested that the extruded nucleus tissue of a herniated disc was infected with bacteria, specifically anaerobic bacteria.^27^, ^28^ There was also a strong statistically significant association between the presence of anaerobic bacteria in vertebrae and the development of Modic changes (MCs) (i.e., subchondral non‐neoplastic vertebral lesions), which are highly associated with disc degeneration. This association between infected disc material by anaerobic bacteria and disc degeneration as evidenced by MCs prompted further exploration into the spine‐gut axis. Rajasekaran et al.^29^ demonstrated disc microbiome dysbiosis between normal and degenerative discs, further bringing forth a spotlight on bacterial etiology as related to disc changes and the role of microbiota.

A plausible explanation for the bacterial colonization seen may include the disc herniation allowing a suitable environment for anaerobic bacteria to travel to the avascular, anaerobic disc and facilitate systemic inflammation while potentiating the inflammation occurring from the herniation.^26^ This cycle of systemic inflammation from the gut bacteria and localized inflammation occurring in the intervertebral discs and perhaps the facet joints may contribute to the development of LDS.^30^

Although the mechanism underlying interrelations between gut microbiota and spinal health are not yet completely elucidated, gut microbiome dysbiosis may play a role in the development of LDS. Therefore, the purpose of this study was to determine if gut microbiome differences exist between symptomatic patients with and without LDS. Symptomatic patients were utilized since this represents a “real‐world” scenario in clinics and to avoid the potential confounder of pain. Otherwise, differences in pain management exposure with an asymptomatic cohort could lead to confounding effects in comparing microbial communities.

## 2 METHODS

### 2.1 Study design and patient recruitment

The Institutional Review Board at Rush University Medical Center approved the study (ORA# 20100905), and all subjects signed the informed consent form before the study activities were performed. Study procedures were conducted according to the Declaration of Helsinki principles.

A cross‐sectional analysis based on a prospective cohort was performed. Fifty‐one subjects were enrolled in the Rush Omics Spine Study Cohort, all of whom have undergone either microdiscectomy for herniated lumbar disc or lumbar fusion for symptomatic intervertebral disc degeneration between L1‐S1 at a single institution. Female and male subjects between 18 and 80 years were recruited, and consent was obtained preoperatively in the outpatient setting at which surgery was offered. Patients stratified to LDS and non‐LDS were noted anywhere from L1‐S1. Exclusion criteria included spine deformities, metabolic spine conditions, spine infection, spine tuberculosis, malignancy, sickle cell disease, pregnancy, diabetes, recent antibiotic use, joint pain, corticosteroid use, radiotherapy, gastrointestinal, and other arthritic comorbidities. The study questionnaire accounted for diet, sleep hygiene, exercise, alcohol consumption, smoking, medication, bowel action, Bristol stool chart, antibiotic exposure, and infection history.^31^, ^32^, ^33^, ^34^ Patients' demographics and clinical data including age (years), sex, race, and body mass index (BMI) in kg/m^2^ were recorded preoperatively. Patients' pain and disability scores, patient‐reported outcomes measurements information system (PROMIS),^35^ Visual Analogue Pain Scale‐Back (VAS‐Back), Visual Analogue Pain Scale‐Leg, and oswestry disability index^36^ were obtained from the electronic medical record preoperatively and postoperatively at 6 weeks, 3 months, 6 months, and 1 year.

### 2.2 Imaging studies

Patients were required to exhibit radiographic signs of degenerative LDS confirmed by magnetic resonance imaging (MRI). Radiographic standardized standing lumbosacral radiographs assessed spinopelvic parameters, including lumbar lordosis (LL), pelvic tilt (PT), sacral slope (SS), pelvic incidence (PI), and the degree of LDS (Figure 1).^37^, ^38^ 1.5 T MRI scans were obtained preoperatively on each participant for detailed visualization of the lumbar spine, and confirmation of the LDS and severity determination. L1 to S1 lumbosacral levels were evaluated using sagittal T1‐ and T2‐weighted MRI images (Figure 1). For the overall project, different patterns of spinal phenotypes were evaluated, such as disc degeneration, disc herniation, endplate damage/change, MCs, and LDS. For the current study, patients were divided between two groups, LDS and non‐LDS subjects. Based on standing lateral plain radiographs, the Meyerding classification^39^ was used for degenerative LDS grading for each lumbar level, classified as follows: Grade I (0%–25%), Grade II (25–50%), Grade III (75%–75%), Grade IV (75%–100%). For the current study, the Pfirrmann et al.^40^ classification was utilized for each motion segment's disc degeneration classification. A cumulative disc degeneration score was obtained by the cumulative individual motion segment disc scores from L1‐S1, resulting in a range of 5–25.^40^ The presence or not of MCs was also assessed and based on the classification scheme by Modic et al.^41^ An orthopedic surgeon and a research fellow with expertise in imaging analyses performed the assessments, yielding excellent reliability (*k* >0.90), consistent with findings from other studies.^42^, ^43^, ^44^, ^45^

### 2.3 Stool collection, DNA extraction, and high‐throughput DNA sequencing of the gut microbiome

Each study participant collected stool samples at their home residence using a validated collection kit (Feces Catcher and DNA/RNA Shield Fecal Collection Tube, Zymo Research, Irvin, CA, USA) prior to surgery and within 3 months in relation to preoperative imaging assessment. Following transportation to the laboratory, stool samples were stored at −80°C until processing. Genomic DNA was extracted from fecal samples using a chemagic DNA Stool 200 Kit (Revvity) and implemented on a Chemagic 360 device. Prior to purification, samples were subject to bead‐beating on a TissueLyzer II device (Qiagen) for 30 minutes″ at 30 Hz. Bead‐beating was performed twice, with a 2‐minutes rest in between each event.

16S ribosomal RNA gene amplicons for sequencing were generated using a two‐stage PCR protocol similar to that described previously.^46^ Genomic DNA was PCR amplified with primers sIDTP5_515F and sIDTP7_806R (modified from the primer set employed by the Earth Microbiome Project) (EMP; CTACACGACGCTCTTCCGATCTGTGYCAGCMGCCGCGGTAA and CAGACGTGTGCTCTTCCGATCTGGACTACNVGGGTWTCTAAT, respectively—underlined regions represent linker sequences) targeting the V4 region of microbial small subunit ribosomal RNA genes. The primers contained 5′ common sequence tags (sIDTP5 and sIDTP7) that match 3′ sequences present in xGen™ Amplicon UDI primers (IDT). First‐stage PCR amplifications were performed in 10 microliter reactions in 96‐well plates, using repliQa HiFi ToughMix (Quantabio). Genomic DNA input was 1 microliter per reaction. PCR conditions were 98°C for 2 minutes, followed by 28 cycles of 98°C for 10 minutes″, 52°C for 1 minute″ and 68°C for 1 minute″.

Subsequently, a second PCR amplification was performed in 10 μL reactions in 96‐well plates using repliQa HiFi ToughMix. Each well received a separate primer pair containing unique dual indices (i.e., from the IDT xGen™ amplicon UDI primer sets). One microliter of PCR product from the first stage amplification was used as template for the second stage, without cleanup. Two microliters of primer were used per reaction. Cycling conditions were 98°C for 2 minutes, followed by 8 cycles of 98°C for 10 minutes″, 60°C for 1 minute″ and 68°C for 1 minute″. Libraries were pooled, purified using a 0.6X AMPure (Beckman‐Coulter) cleanup, and sequenced with a 10% phiX spike‐in on an Illumina Miniseq sequencer employing a mid‐output flow cell (2 × 154 paired‐end reads). Library preparation, pooling, and sequencing were performed at the Genomics and Microbiome Core Facility at Rush University.

Microbiome bioinformatics were performed with QIIME2.^47^ Raw sequence data were checked for quality using FastQC and merged using the software package PEAR.^48^ Merged sequences were quality filtered using the q2‐demux plugin followed by denoising with DADA2 (via q2‐dada2).^49^ Primer adapter sequences were removed using cutadapt algorithm. Alpha‐diversity metrics and beta‐diversity metrics were calculated using q2‐diversity after samples were rarefied.^50^ Taxonomy was assigned to amplicon sequence variants (ASVs) using the q2‐feature‐classifier classify‐sklearn naïve Bayes taxonomy classifier against the SILVA 138 99% reference sequences database.^51^ The contaminant removal software, decontam, did not detect any contaminants based on the prevalence of the ASVs in the reagent‐negative blank controls using default parameters.

### 2.4 Statistical analyses

Data analyses were performed using R version 4.0.3 (RRID: SCR_000432, RRID: SCR_001905). The microbial abundance data from the read count table were transformed into Phyloseq objects (RRID: SCR_013080). Alpha diversity indices (i.e., Evenness, Shannon index, Simpson index, and Chao1) were compared between participants with or without LDS using the microbiome package (RRID: SCR_024699).

Beta diversity (between‐sample) analyses (Bray–Curtis dissimilarity) were performed at the taxonomic level of genus on a filtered dataset to remove features present in fewer than half the samples at a depth of fewer than 5 reads. Statistical evaluation of differences in total microbial community between groups was using permutational MANOVA (PERMANOVA) with the adonis2 function of the vegan R package (RRID: SCR_011950). The analysis was repeated while adjusting for age and BMI.

To assess the impact of confounding variables on the diversity of the microbiome, we conducted a redundancy analysis using the rda function from the vegan package. The covariates we considered were LDS, age, BMI, and sex. Data were filtered based on prevalence of >10% and were then subjected to Hellinger transformation prior to executing the rda function. Subsequently, we employed an environmental fit model by utilizing the envfit function from the vegan package. This was done to examine the spatial impact of LDS while considering any potential confounding factors.

The ratio of Bacillota (formerly Firmicutes) to Bacteroidota (formerly F/B ratio) was computed on the transformed relative abundance data as previously mentioned. Differences were then assessed among subjects with LDS versus non‐LDS using the Mann–Whitney test. The analysis was replicated utilizing a linear regression model to account for age, BMI, and sex. The formula used was (F/B ratio ~ LDS + age + BMI + sex). To investigate the differential relative abundance of the microbial taxa among subjects with and without LDS, we employed Analysis of Compositions of Microbiomes with Bias Correction 2 (ANCOM‐BC2) analysis at the taxonomic level of genus.^52^ Based on the significance criterion, which comprised a *p*‐value<0.05 and a *q‐*value<0.25 to control the false‐discovery rate, the significant features were chosen. The figures were generated in R using the dplyr (RRID: SCR_016708), ggplo2 (RRID: SCR_014601), and ggpubr (RRID: SCR_021139) packages.

Raw sequence data files were submitted to the sequence read archive of the National Center for Biotechnology Information under the BioProject identifier PRJNA1099668.

## 3 RESULTS

This study included a total of 33 subjects, comprising 12 (36.4%) patients without LDS and 21 (63.6%) patients with LDS (Table 1). Nineteen (57.6%) subjects were males and 14 (42.4%) were females. The mean age of the cohort was 57.7 ± 13.9 years. Patients with LDS had a mean age of 61.9 ± 8.1 years as compared to those without LDS (mean age of 50.3 ± 18.7 years; *p* = 0.09). Although not statistically significant, there was a higher prevalence of females among those with LDS; 78.5% of the female subjects have LDS compared to 52.6% of male subjects (*p* = 0.24). No significant differences were observed in BMI, smoking, or alcohol consumption between patients with and without LDS (*p* >0.05). Pain and disability scores did not differ between the LDS and non‐LDS groups (*p* >0.05). Table 1 also notes the surgical procedures between groups. As expected, the rates of fusion were higher in the LDS group and attributed to the nature of the disorder and established management principles (*p* = 0.001).

**TABLE 1:** Patient demographics and clinical parameters in relation to the presence or not of spondylolisthesis of the lumbar spine in patients.

Variables	Non‐LDS	LDS	Overall	*p*‐value
	(*n* = 12)	(*n* = 21)	(*N* = 33)	
Age				
Mean (±SD)	50.3 (18.7)	61.9 (8.06)	57.7 (13.9)	0.089
Gender				
Female	3 (25.0%)	11 (52.4%)	14 (42.4%)	0.240
Male	9 (75.0%)	10 (47.6%)	19 (57.6%)	
Race				
Non‐White	4 (33.3%)	6 (28.6%)	10 (30.3%)	1
White	8 (66.7%)	15 (71.4%)	23 (69.7%)	
BMI				
Mean (±SD)	29.1 (5.69)	28.3 (5.24)	28.6 (5.34)	0.760
Median[Min, Max]	27.7 [21.0, 39.1]	27.1 [22.5, 41.3]	27.1 [21.0, 41.3]	
Smoker				
Current	1 (8.3%)	0 (0%)	1 (3.0%)	0.632
Former	3 (25.0%)	6 (28.6%)	9 (27.3%)	
Never	8 (66.7%)	15 (71.4%)	23 (69.7%)	
Alcohol				
Daily	2 (16.7%)	4 (19.0%)	6 (18.2%)	0.451
Never	4 (33.3%)	8 (38.1%)	12 (36.4%)	
Socially	2 (16.7%)	0 (0%)	2 (6.1%)	
Weekly_3 or >	3 (25.0%)	2 (9.5%)	5 (15.2%)	
Weekly once	1 (8.3%)	3 (14.3%)	4 (12.1%)	
Previous	0 (0%)	1 (4.8%)	1 (3.0%)	
Weekly_2 drinks	0 (0%)	3 (14.3%)	3 (9.1%)	
Surgical Management				
Discectomy	2 (16.7%)	0 (0%)	2 (6.1%)	0.001
Laminectomy	0 (0%)	2 (9.5%)	2 (6.1%)	
Discectomy/Laminectomy	8 (66.7%)	2 (9.5%)	10 (30.3%)	
Fusion	2 (16.7%)	17 (81.0%)	19 (57.6%)	

Abbreviations: BMI, body mass index; LDS, lumbar degenerative spondylolisthesis; SD, standard deviation; Min, minimum; Max, maximum.

### 3.1 Imaging phenotypes

A total of 50 LDS conditions were identified in 21 patients. These LDS were categorized into two types: retrolisthesis (*n* = 26) and anterolisthesis (*n* = 24). Ten patients exhibited both types of LDS in their imaging results. Among the different lumbar levels, the L4‐L5 and L5‐S1 segments (*n* = 14/each segment) demonstrated the highest prevalence of LDS. Such prevalence rates of these segments were followed by the L2‐L3 (*n* = 11), L3‐L4 (*n* = 7), and L1‐L2 (*n* = 4). Retrolisthesis LDS, which was the most frequently observed subtype, was present at each level, with the highest occurrence at the L2‐L3 (*n* = 7) and the L3‐L4 (*n* = 5) levels. Grade 1 LDS was the most common phenotype with 41 cases (82%) observed, followed by Grade 2 with 7 cases (14%), and Grade 3 with 2 cases (4%). There was a statistically significant difference in disc degeneration score between the LDS and non‐LDS groups. LDS individuals had a score of 16.3 ± 3.5 whereas non‐LDS subjects had a mean disc degeneration score of 12.8 ± 3.9 (*p* = 0.018). In addition, there was no statistically significant difference between the presence or not of MCs between groups (*p* = 0.741) (Table 2).

**TABLE 2:** Summary of imaging phenotypes in relation to the presence or not of spondylolisthesis of the lumbar spine in patients.

Imaging phenotypes	Non‐LDS (*n* = 12)	LDS (*n* = 21)	Overall (*N* = 33)	*p*‐value
Lumbar lordosis	47.7 ± 10.4	48.4 ± 18.1	48.1 ± 15.5	0.454
Pelvic tilt	15.6 ± 7.8	18.8 ± 7.9	17.6 ± 7.9	0.178
Sacral slope	34.7 ± 4.0	36.9 ± 8.0	36.1 ± 6.8	0.852
Pelvic incidence	49.5 ± 10.0	55.7 ± 9.9	53.5 ± 10.3	0.155
PI‐LL mismatch	1.8 ± 14.9	7.3 ± 14.6	5.3 ± 14.7	0.626
Disc degeneration score	12.8 ± 3.9	16.3 ± 3.5	15.0 ± 3.9	0.018
Modic changes				
No	7 (58.3%)	11 (52.4%)	18 (54.5%)	0.741
Yes	5 (41.7%)	10 (47.6%)	15 (45.5%)	

Abbreviations: PI, pelvic incidence; LL, lumbar lordosis..

Table 2 summarizes the sagittal alignment characteristics in patients with and without LDS. There were no significant differences observed in LL, PT, SS, or PI between the two groups (*p* >0.05). Additionally, no significant between‐group differences in the PI‐LL mismatch were found (*p* = 0.626). These findings suggest that variations in sagittal alignment parameters, in the context of the current study cohort, did not significantly differ between patients with and without LDS.

### 3.2 Gut microbiome diversity: Variations among individuals with and without spondylolisthesis

**FIGURE 2:**
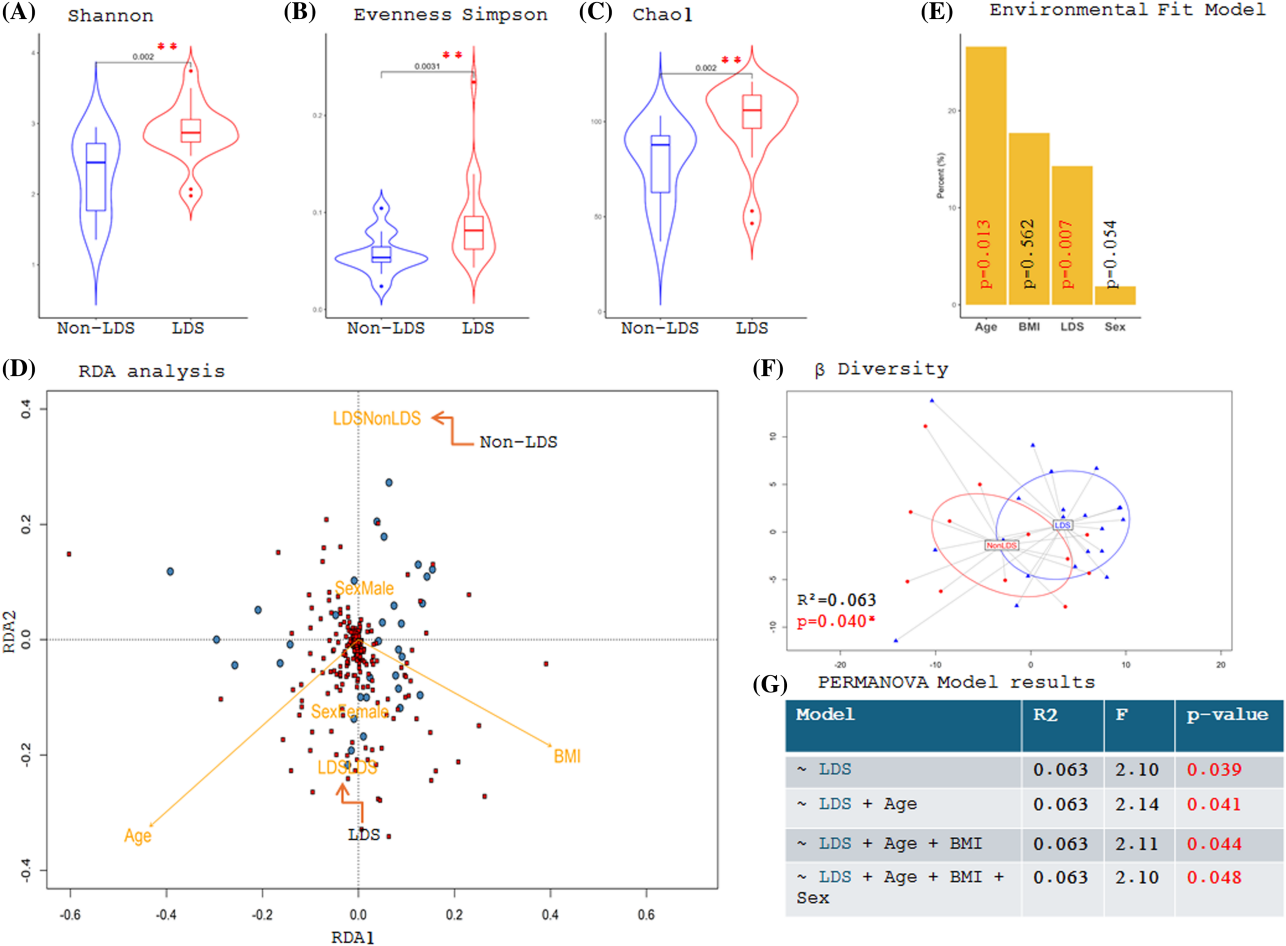
Analysis of fecal microbial community structure using 16S rRNA gene amplicon sequencing. Comparison of alpha diversity indices between groups, including (A) Shannon‐index, (B) evenness, and the parametric richness estimator, Chao1. (D) RDA analysis comparing fecal microbiomes of LDS participants and non‐LDS participants. (E) Environmental fit model with LDS, age, BMI and sex as factors. (F) Ordination of the total microbial community using PCA. (G) PERMANOVA model results accounting for LDS, age, BMI and sex. BMI, body mass index; LDS, lumbar degenerative spondylolisthesis.

The gut microbiome findings revealed significant differences in alpha diversity between groups, with LDS subjects exhibiting higher Shannon indices, Evenness Simpson, and Chao1 compared to subjects without LDS (Figure 2A–C). We next performed analysis to analyze gut compositional differences in microbial communities based on LDS. At first, we investigated the correlation between LDS and established confounding covariates such as age and BMI, and their impact on the microbiome composition. The tested covariates showed a statistically significant spatial effect on the microbial diversity (Figure 2D, E) independent of each other. Subjects with LDS had a spatial direction that correlated with increasing age and BMI, as evidenced by the clustering of LDS at the lower end of the bi‐plot, aligned with the direction of age and BMI. Following, we examined β diversity to characterize differences in gut microbial community structure between participants with and without LDS. Small, but significant differences between groups were observed (PERMANOVA *r* ^2^ = 0.063, *p* = 0.040; Figure 2F, G; taxonomic level of genus). The observed differences were significant even after controlling for confounding variables, such as age (*r* ^2^ = 0.063, *p* = 0.041), BMI (*r* ^2^ = 0.063, *p* = 0.044), and sex (*r* ^2^ = 0.063, *p* = 0.048) (Figure 2G).

### 3.3 Differential abundance of gut microbiota in spondylolisthesis and taxonomic patterns

**FIGURE 3:**
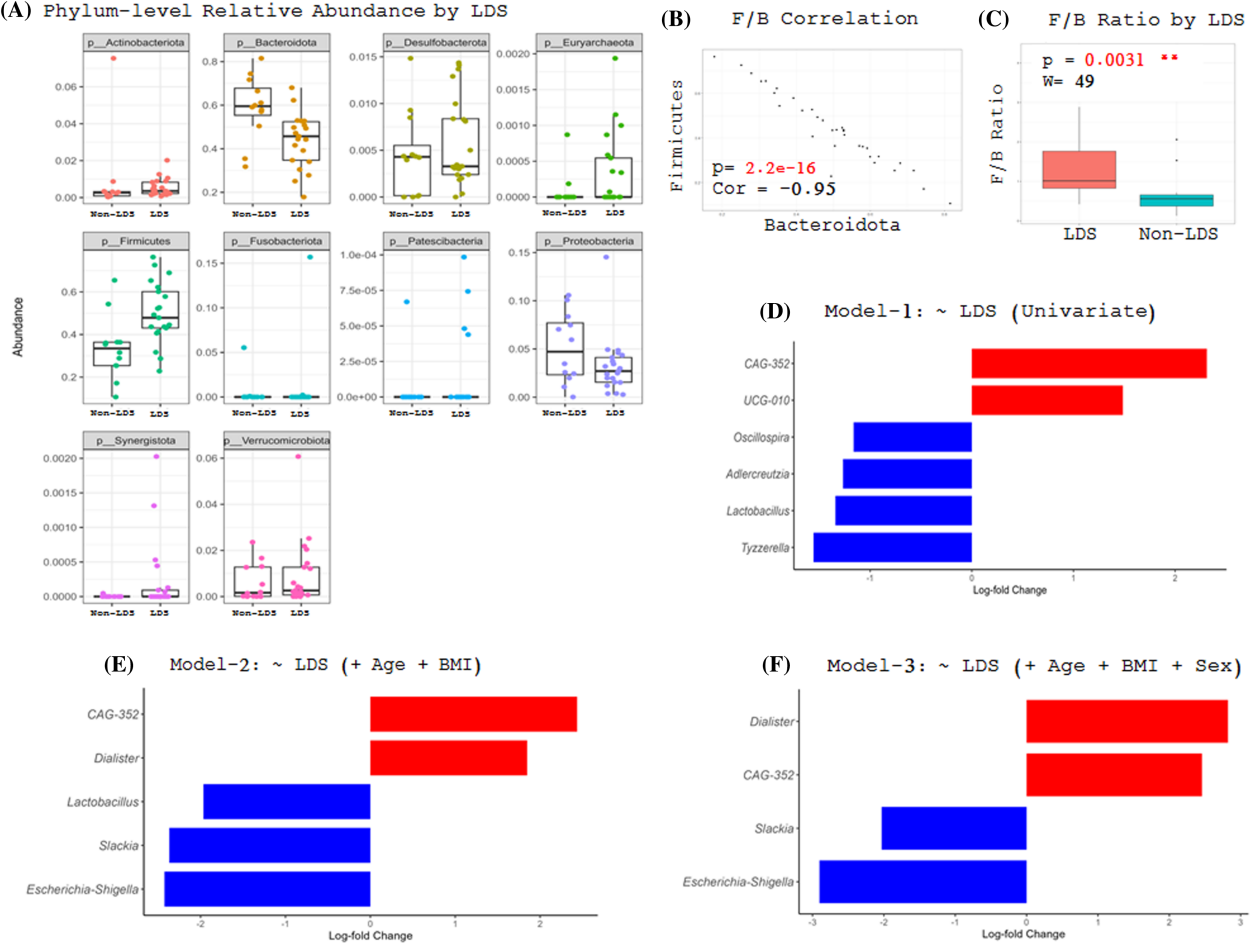
Microbial taxa associated with the presence or absence of LDS. (A) Phylum‐level relative abundance. (B) Firmicutes to Bacteroidota correlation. (C) Firmicutes/Bacteroidota ratio. (D) Model: ~ SL (Univariate). (E) Full Model: ~ LDS (+Age + BMI). (F) Full Model: ~ LDS (+Age + BMI + SEX). BMI, body mass index; LDS, lumbar degenerative spondylolisthesis.

The differential abundance at the phylum level differed between subjects with and without LDS (Figure 3A). The relative abundance of Firmicutes and Bacteroidota exhibited an inverse relationship, indicating that higher levels of Bacillota (formerly Firmicutes) correlated to lower levels of Bacteroidota, and vice versa (Figure 3B). The ratio of Bacillota to Bacteroidota (formerly F/B ratio) was considerably higher in patients with LDS versus non‐LDS (*p* = 0.0031, Figure 3C). We repeated the analysis considering the covariates of age, BMI, and sex, and the analysis remained statistically significant (Estimate: −0.7, *T* = −2.19, *p* = 0.037).

To assess the differential abundance of each taxon between LDS and non‐LDS participants, we ran a regression model using ANCOM‐BC2 package at the taxonomic level of the genus. A total of six differentially abundant taxa were identified. Subjects with LDS had higher relative abundance of putative pro‐inflammatory bacteria (*Clostridium CAG‐352* and *Ruminococcaceae UCG‐010*) and lower levels of putative anti‐inflammatory beneficial bacteria (*Oscillospira*, *Adlercruetzia*, *Lactobacillus*, *Tyzzerella*; Figure 3D). We repeated the model while adjusting the covariates of age, BMI, and sex. Using this model, four taxa were significantly different between groups. The relative abundance of *Dialister* and *CAG‐352* was significantly higher in individuals with LDS. The full model also revealed high relative abundance of putative pro‐inflammatory bacteria from the genus *Dialister* and lower relative abundance of bacteria from the genera *Sackia* and *Escherichia‐Shigella* (Figure 3E,F).

## 4 DISCUSSION

This is the first study, to our knowledge, to demonstrate differences in gut microbial community structure among surgical patients who exhibited evidence of LDS as compared to symptomatic patients without LDS. Fecal microbiome characterization, performed using 16S rRNA gene amplicon sequencing, identified differences in alpha diversity between groups as well as in community structure between groups (beta diversity). These differences were maintained after adjusting for age and BMI. On phylum‐level abundance analysis, Bacillota (formerly Firmicutes) and Bacteroidota demonstrated an inverse relationship across LDS and control subjects, and the Firmicutes:Bacteroidetes ratio was significantly higher in the LDS group. At the genus level, subjects with LDS exhibited higher levels of *Dialister* and *CAG‐352*, while showing reduced presence of *Slackia* and *Escherichia‐Shigella* as compared to those without LDS, after adjusting for age, BMI, and sex. Furthermore, overall disc degeneration severity of the lumbar spine was significantly higher in the LDS group as compared to the non‐LDS subjects.

As lifespan and the prevalence of chronic disease continue to rise in the United States, the influence of factors such as gut microbiome composition on health becomes increasingly significant for comprehending disease treatment and prevention. The bacteria in the gut have numerous roles aiding in metabolism, protective barrier activity, vitamin and bile acid homeostasis, short‐chain fatty acid production, digestion, and immune modulation.^53^ Disruption of these functions through altered bacterial composition and abundance, termed “dysbiosis” has been linked to many disease states in distant organ sites. Because of the vast diversity of the gut microbiome, many factors such as age, diet, genetics, disease, and geographic region have impact on gut microbiome ecology.^54^, ^55^ Advancements in next‐generation sequencing technology, among other efforts such as the Human Microbiome Project, have significantly aided endeavors to reveal the interplay of the gut microbiome with distant organs, most notably exemplified by the “gut‐brain axis.”^56^ While tremendous strides have been made in linking the microbiome to disease, there exist several inherent challenges associated with microbiome research, including high inter‐individual variability, reliance on relative abundance metrics, and dependence on fecal samples.^55^

Autoimmune diseases, such as multiple sclerosis, have been linked to gut dysbiosis, with pediatric multiple sclerosis patients showing a significant reduction in short‐chain fatty acid‐producing *Ruminococcaceae* and *Anaerosporobacter* compared to healthy children.^57^ Bacteriocins synthesized by gut microbiota keep commensal pathogens like *Clostridium difficile*, *Campylobacter jejuni*, *and Salmonella species* in healthy numbers. Once gut dysbiosis is introduced, the risk of colorectal cancer is greatly increased with the reduction in bacteriocins.^58^ Neuropsychiatric disorders including depression, schizophrenia, bipolar disorder, and autism spectrum disorder can be linked to gut dysbiosis through the gut‐brain axis.^59^ Altered microbial composition and metabolites altering the levels of neurotransmitters, and the development of the leaky gut through decreased tight junction proteins allowing for central nervous system penetration facilitates a pro‐inflammatory state leading to the development of various neuropsychiatric disorders.^59^ The multifactorial nature of gut dysbiosis lends to its systemic wide implications in the human body, ranging from autoimmune diseases to neuropsychiatric diseases. The leaky gut‐altering microbiome composition allows for systemic spread throughout the body providing a basis for its role in disease.

While little is known about the interaction of the human microbiome and musculoskeletal diseases, some literature has emerged in the field over the past couple of decades. In a case‐control study by Cardoneau et al.^24^ assessing fecal samples via real‐time polymerase chain reaction, ankylosing spondylitis patients showed decreased bacterial diversity compared to controls. As bacterial processes are highly linked to bone health function, various studies have explored the role of the gut microbiome in bone density and osteoporosis.^23^, ^60^, ^61^, ^62^, ^63^ The microbiome has also been implicated in rheumatoid arthritis (RA), with RA subjects showing an increased relative abundance of bacteria from the genus *Prevotella*.^64^, ^65^ The microbiome has also been studied in sarcopenia, a disease characterized by age‐related skeletal muscle mass reduction, showing reduced microbial diversity and increased pro‐inflammatory markers in sarcopenic patients.^66^ In the progression of osteoarthritis, some studies suggest that a bacterial‐derived inflammatory etiology plays a role in the disease.^66^, ^67^, ^68^, ^69^

While there is a paucity of literature surrounding the interplay between microbiota and musculoskeletal health, there are even fewer studies involving spinal pathologies, such as disc degeneration, MCs, and facet joint degeneration, processes associated with degenerative LDS and low back pain. In a study of overweight and obese patients with and without back pain, subjects with back pain had higher relative abundance of the putatively anti‐inflammatory bacteria from the genus *Adlercreutzia*, also observed in our study.^25^ The study also noted that the relative abundance of *Adlercreutzia* was correlated with BMI. While there are currently no studies focusing on the facet joint and the microbiome, new research has investigated subclinical infection in the intervertebral discs.^27^, ^70^, ^71^, ^72^, ^73^ Similar to the blood–brain barrier, a blood‐disc barrier exists that protects the disc from systemic inflammation and infection.^74^ While discs were previously thought of as sterile, there is growing evidence that the environment of the disc can facilitate growth of anaerobic bacteria.^29^ Infection‐mediated release of inflammatory cytokines, such as IL‐6 and TNF‐alpha, could further damage the disc, leading to disc degeneration and a pro‐inflammatory environment. MCs, a well‐defined imaging parameter of vertebral bone marrow, have been linked to the development of low back pain.^75^, ^76^, ^77^, ^78^, ^79^, ^80^, ^81^, ^82^ Recent studies have indicated that MCs are associated with low‐grade infections in the intervertebral disc.^26^, ^71^, ^83^, ^84^, ^85^, ^86^, ^87^, ^88^, ^89^, ^90^ Furthermore, Albert et al.^91^ and Wong et al.^92^ reported improvement in low back pain symptoms associated with Type 1 MCs following antibiotic treatment. However, it should be noted that in our current study, there was no statistically significant difference between the rates of MCs between patients with or without LDS; however, assessing and analyzing Modic types in this context due to the sample size of the study presented a challenge that hopefully future, larger‐scale studies can address. Rajasekaran et al.^29^ examined genomic DNA from 24 lumbar intervertebral discs in brain‐dead yet alive donors and reported that healthy disc microbial composition differed significantly from herniated and degenerated discs, demonstrating that the disc may contain a microbiome similar to that of the gut. Li et al.,^93^ in a prospective literature review, identified three potential mechanisms for microbiota introduction into the disc: (1) permeability of the gut epithelial barrier, (2) immune dysregulation, and (3) nutrient absorption and metabolite formation. Su et al.^94^ was one of the first to utilize Mendelian randomization analysis to establish a causal relationship between microbiota and low back pain. They reported microbial phyla with positive causal relationships with development of low back pain. Geng et al.^95^ further investigated this causal relationship, specifying directionality of the association. Utilizing a two‐sample Mendelian randomization study, the authors noted a unidirectional association between the *Bacteroidetes* phylum and the development of intervertebral disc degeneration, with no evidence of disc degeneration affecting the gut microbiota.^95^ Despite being a relatively new concept in the field of musculoskeletal research, there has been growing literature on the association between the gut microbiome and the spine, providing evidence for a “gut‐spine axis.”^96^

LDS has a vast history with Kilian proposing the term “spondylolisthesis” and describing it as a slow displacement of the lumbar vertebrae.^5^ In time, this definition shifted toward describing LDS solely being caused by a pars interarticularis defect, with other etiologies being termed pseudospondylolisthesis.^5^ With advancements in technology, various classification schemes of “spondylolisthesis” came to light. Newman and Stone^10^ were the first to report the term “degenerative spondylolisthesis” in 1955, describing it as the slippage of the vertebrae with an intact neural arch. This could be attributed to facet joint degenerative arthritis, differentiating it from previously known classifications of isthmic or dysplastic LDS. In their classification scheme, there was no pars interarticularis defect as was proposed prior, but instead, the condition was attributed to long‐standing disc degeneration causing the vertebral body to slip.

LDS can often present with various other spinal comorbidities, such as osteoarthritis, spinal stenosis, and disc degeneration, making its etiology difficult to determine.^14^ Despite its multifactorial nature and as previously mentioned, LDS is more commonly encountered in older adults (age >50), females with a 6 to 1 prevalence, and can often be localized to the L4‐L5 vertebral segment.^7^, ^8^, ^9^, ^10^, ^11^ This can be differentiated from the isthmic classification scheme where the L5‐S1 vertebral level is more commonly affected. A plausible explanation for the difference in lumbar spinal levels could be due to the strong iliolumbar ligaments that keep L5 in its anatomical position, and the movement that is generated through the lumbar spine is the largest at the L4 level predisposing it to degeneration.^14^ The most common complaint of patients with LDS is low back pain, a major health problem globally. Other common complaints include radiculopathy and neurogenic claudication from possible spinal stenosis.^97^

Facet joint arthritis and disc degeneration have been linked to the development of degenerative LDS. The progressive loss of cartilage and continuous articular remodeling that occurs in osteoarthritis can allow for weakening of the anterior restraint supplied by the facet joints. Fibrosis, angiogenesis, and inflammatory cells predominate in the joint capsule during the arthritic stage and contribute to the weakening of the facet joint.^98^ Other common inflammatory conditions that affect the facet joints include rheumatoid arthritis, ankylosing spondylitis, synovial inflammation, and acute and chronic infection prompting the discussion of inflammation and inflammatory markers in the pathogenesis of facet joint degeneration.^99^

### 4.1 Gut microbiota

In our study, abundance analysis of taxa in LDS subjects determined higher levels of *Dialister* and *CAG‐352*, when controlling for age, BMI, and sex. Bacteria from the taxa *CAG‐352* and *Dialister*, members of the phylum Bacillota, have been studied in numerous diseases and conditions.^59^, ^100^, ^101^, ^102^, ^103^, ^104^, ^105^, ^106^ Concerning musculoskeletal disease, Song et al.,^107^ based on a non‐peer‐reviewed pre‐print, studied ankylosing spondylitis patients and found *CAG‐352* abundance, in association with *Agathobacter*, *Ruminococcus*, and *Prevotella*, positively correlated with lymphocyte subsets (B‐cells, Th1 cells) and cytokine levels. In a study by Tito et al.,^108^ the relative abundance of bacteria from the genus *Dialister* was positively correlated with ankylosing spondylitis disease activity. In patients with rheumatoid arthritis, Mena‐Vazquez et al.^109^ analyzed the gut microbial community structure and observed a correlation of the relative abundance of *Dialister* with increased obesity and fat mass index.

The Bacillota and Bacteroides families, which generally represent approximately 80% of the fecal taxa observed in microbial community analyses, have been implicated in a wide array of diseases and have garnered attention in the literature.^110^ The Bacillota:Bacteroidetes (formerly F:B) ratio is commonly cited in microbiome literature as a marker for obesity, but can also be used as a proxy for shifts in overall microbial community structure.^111^, ^112^ However, there is controversy in the literature regarding this claim, as many of the studies reporting an increased F:B ratio in obesity also demonstrated overall lower microbiota diversity in obese subjects.^113^ Increases in the F:B ratio have also been linked to the development of prostate enlargement and inflammation giving way to various urologic pathologies.^114^ With a prominent role in the development of various systemic pathologies, there exists a necessity for literature on exploring these gut‐organ systems axes. While there is no current literature surrounding the F:B ratios in feces of patients with spine diseases, our study is the “first‐of‐its‐kind” to report an increased F:B ratio in patients with LDS, when controlling for age, BMI, and sex. It is also noteworthy that an increase in lumbar disc degeneration severity was noted in subjects with LDS in comparison to non‐LDS subjects, implicating the disc as a mediator/pathway of gut microbiome dysbiosis. Although not assessed in our current study, the impact of such dysbiosis upon “facet joint changes” (a hallmark of LDS) is a viable concern and needs future assessment. In fact, studies have noted that patients with LDS had a significantly higher level of pro‐inflammatory markers and receptors in the facet joints and, to a lesser degree, the disc.^115^ Therefore, the impact of the gut microbiome may exert its effects upon the disc, facets, or both. It should also be noted that the F:B ratio is known to be significantly higher by almost four‐fold in females than males.^116^ As the LDS group had more females than the non‐LDS group, the higher F:B ratio might be partly attributed to more females in the LDS group “but” may also partly explain the higher prevalence in populations of females with LDS as an attribute of the gut microbiome profile. Further research is needed to investigate the specific pathway how gut dysbiosis leads to LDS‐related degenerative changes.

### 4.2 Strengths and limitations

The present study contains inherent limitations representative of most microbiome‐related studies. The first primary limitation is a high inter‐individual variability between the microbiomes of individuals. While microbiome data can be highly variable, in this study there were no observed differences in covariates like BMI, age, smoking, alcohol consumption, and presence of pain between groups, and any analyses that adjusted for covariates with known influences on microbiome composition such as age, BMI and sex. Second, the study of 16S rRNA genes is limited to taxonomic identification at the genus level, hence lacking precise information pertaining to species‐level bacterial classification, and may not thoroughly distinguish between active to that of inactive microbiota. Similarly, measurement bias by surgeon assessment of LDS is another systematic error that could have impacted results. To mitigate this, only two highly trained reviewers, an orthopedic surgeon, and a research fellow performed the assessments with excellent reliability (*k* >0.90). Furthermore, although degenerative spinal conditions evolve over time, the rate of this development is dependent over many factors in the risk profile of whatever degenerative phenotype is being studied on imaging. There may be triggers of degeneration rate increase or regression of that rate based on many factors, inflammatory profile, genetics, and aging in general being of some key mediators.^32^, ^34^, ^80^, ^117^, ^118^, ^119^ It is widely acknowledged that the intestinal microbiota exhibits an increasingly pro‐inflammatory “inflammaging” profile with increased numbers of gut bacteria with aging.^120^, ^121^, ^122^ In short, the gut microbiome/microbiota profile is relatively stable day to day but steadily more marked inflammation with aging. In addition, due to the study design, making robust conclusions related to causality is a challenge. Nonetheless, this work raises awareness and provides empirical evidence of an association between the spine‐gut axis that warrants future prospective study.

## 5 CONCLUSION

In conclusion, to our knowledge, our study is the “first” to explore the relationship between gut dysbiosis and the development of LDS. The study results noted a higher Bacillota (formerly Firmicutes) to Bacteroides ratio in patients with symptomatic LDS. The relative abundance of bacterial taxa *CAG‐352* and *Dialister* (within the phylum Bacillota) was significantly higher in patients with LDS. Although larger, multicenter studies are needed to validate our results, the relationship between gut dysbiosis and LDS may provide a paradigm shift in terms of the etiology of spondylolisthesis. Such findings may lend credence to an inflammatory, microbial, metabolic, or integrative platform related to LDS. In addition, these findings may in time lend to in‐depth personalized treatment options and predictive modeling for at‐risk patients and usher forth novel, targeted therapeutics taking into account gut health.

## FUNDING INFORMATION

National Institutes of Health, National Institute of Arthritis and Musculoskeletal and Skin Diseases, Grant # R21AR079679, and the Thomas J. Coogan Sr., MD, Chair of Immunology Endowment.

## CONFLCT OF INTEREST STATEMENT

The authors have no financial or conflicting interests to disclose.
